# Reflections on Science and Ethics*

**DOI:** 10.5041/RMMJ.10012

**Published:** 2010-10-31

**Authors:** Elie Wiesel

**Affiliations:** Nobel Prize Laureate in Peace, 1986. Andrew W. Mellon Professor in the Humanities; Professor of Philosophy and Religion, Elie Wiesel Center for Judaic Studies, Boston University, Boston, USA. Distinguished Presidential Fellow at Chapman University, Orange, California, USA

Can science be ethical? Is it supposed to be ethical?

I am a student of philosophy. In the beginning I had actually thought of studying music, not so much because I love music – but because music has never caused war. Only words produce war, and, having become afraid of words, I thought – why not study music and become a conductor? But then I chose philosophy – because of the *questions it involves*; and I almost left philosophy – because of the *answers*. Now, the questions that you ask, that all of you ask, are actually mystical answers. All the things you heard during this Symposium have been approached, studied, communicated, sometimes without words, but within mysticism. The origin of the universe, the darkness, even the end of time: it is all mysticism. You scientists, whom I admire, have given us a view of how the universe is constituted. However, in my view, there is one issue that you all, willingly or not, or consciously or not, have not addressed: “*Why is there an universe?*”

In *mysticism* we know why God created the world. As for ethics, we ask ourselves – is the universe concerned with ethics? All these electrons, molecules, are they at all concerned with ethics?

Reading the Bible, we learn of the fateful situation that evolved after our grandmother Eve ate the forbidden fruit: it was the beginning of good and evil. The Bible says that at that very moment Adam and Eve also realized the difference between good and evil.

We are currently told that this huge universe irrupted, my God, billions of years ago. Be it 17 billion or 300 million years, at 3 o’clock in the afternoon – all of a sudden the universe came into being! Hegel already asked: “What did God do before he created the world?” What will we do after this event? I regard this to be an ethical question.

I am also a student of history. I love history, firstly because of my Talmudic and Hassidic background. In the Talmud, the question that is all the time there is what we call *Ma Ko Mashma Lan*, namely “what do we learn from this” – in other words: what does it mean?

OK, we do realize that there is a universe, but – what does it mean? There is darkness, what does it mean? What is the meaning of the existence of the universe?

Secondly, in Jewish tradition we have always been reluctant to love history. We didn’t fare well with history. Our early historians, like Josephus Flavius, we didn’t like because they were too close to the Romans. Josephus was a brilliant Jewish general, who gave himself up to the Romans – seduced by the luxury and by the power of Rome. He became a spokesman of Rome to the Jews rather than being a spokesman of the Jews to Rome. We were hardly taught about Josephus, although he is coming back into the curriculum.

Then we had the historian Heinrich Gretz in Germany, but I personally do not like him because he literally hated Hassidism. A more recent historian, Shimon Dubnow, did justice to Hassidism. He loved Hassidism. One day I looked up something about him in the *Encyclopedia Judaica*, and I found that Shimon Dubnow had for years taught in Germany and moved later on to Latvia, to Riga, where he was forced to live in the ghetto. On 7 or 9 December 1941, Dubnow was killed by a former student of his. Who was this student? Why did he murder Dubnow? This was the puzzle I was working on, searching through all possible sources. I finally discovered that Dubnow was killed by the head of the Gestapo in Riga and that this man, named Dr Johann Silber, had been his student. And then I learnt that this Johann Silber used to come to the ghetto just to taunt Dubnow, to ridicule him, saying, “Professor are you still a humanist?” Dubnow answered, “Yes”. “Do you still believe in the human condition?” “Yes”, Dubnow said. And then Silber said: “Last week we executed, with my participation, 700 Jews. And you still declare that you believe in human nature?” And Dubnow took out his pencil and paper: “Exactly how many Jews did you say you executed?”

He remained a historian to the end!

And I said to myself, my God, this Johann Silber had a Doctor’s degree from a prestigious German university! As you perhaps know, a doctorate in Germany in those times was much more difficult to achieve than it is at present in America or here in Israel. So, what had happened? How is it possible that education proved to be no barrier to barbarism? That cultured people did not stop Silber from doing what he did?

We know from history the occasional outburst of winds of madness. The Crusades were madness, absolute madness. The Inquisition was madness, and, in a certain way, even Hitler and the Final Solution were madness. And today, if we do not proceed very carefully, the spread of fanaticism in the world, with fanaticism gaining power in diverse places – might evolve into evil madness, destructive madness. A similar phenomenon could happen to science: science could simply lose its ethical compass.

The first professionals to join Hitler’s Nazi programs were physicians. Medical doctors performed the “euthanasia plan”. We also learned what went on in the concentration camps, where cruel “medical” and “surgical” experiments were imposed coercively on helpless inmates. Professors in medicine did such research on human subjects, without anesthesia, perhaps with the pretext that it was for the benefit of science and of mankind.

This position, by the way, presents us with an ethical dilemma. I was asked years ago, whether I thought that it would be right to adopt and apply useful data and results that can be derived from those barbaric experiments. May we use them? And, whereas according to law one cannot use documents that have been obtained illegally, what should be our standing in this respect regarding scientific knowledge thus obtained?

As a “coda” to my talk, I will again address material that I found in documents on Dubnow. Dubnow, who, as we know, was in the ghetto. And he was writing his “History of the Jewish People” but did not finish it. The final chapter of this monumental work was written in the ghetto, but it vanished. We do not know where it is, and I am sure it must be hidden somewhere, just like the original diaries we found in milk cans in Warsaw and others that were concealed in the Sonderkommando quarters in Birkenau. Those we managed to find, and one day we will also find Dubnow’s writings.

But here is what I *did* come upon. In the very last volume that he managed to get published Dubnow writes: “At the end of my life I would like to return to my native town in White Russia. I have not seen it in some 30 years. I would like to go to the cemetery, visit the grave of my grandfather, the great Rebbe ben Zion, and whisper to him, ‘It’s me, your grandson Shimon. I have come here, being at the age you were when you left this world. Do you remember my rebellion against Judaism? My hostility toward all things you considered sacred? Do you recall it? The sadness you felt when I left you and your way of life, do you remember it, Grandfather? You told me then, that one day I would return to the source which I rejected because I viewed Judaism as a caricature, do you remember it Grandfather? Well, Rebbe ben Zion, my grandfather, see – your prophecy has been fulfilled’.” But Dubnow did not return. He was murdered in the ghetto by a student of his, Johann Silber, “who had a doctorate”.

Therefore, my dear colleagues, please be aware that science, with all its greatness, with all its depth, and with all the fascinating vision that you own about universe and its galaxies, is not sufficient if it stands by itself. Please remember, that while the mystery of the human being may reside in the galaxies, it lies even more so in the human heart.

